# Feasibility and impact of inverted classroom methodology for coronavirus disease 2019 (COVID-19) pandemic preparedness at an urban community hospital

**DOI:** 10.1017/ice.2020.1272

**Published:** 2020-10-20

**Authors:** Alfredo J. Mena Lora, Mirza Ali, Candice Krill, Scott A. Borgetti, Sherrie Spencer, Romeen Lavani, Eden Takhsh, Susan C. Bleasdale

**Affiliations:** 1 Division of Infectious Diseases, Department of Medicine, University of Illinois at Chicago, Chicago, Illinois; 2 Saint Anthony Hospital, Chicago, Illinois

## Abstract

Strategies for pandemic preparedness and response are urgently needed for all settings. We describe our experience using inverted classroom methodology (ICM) for COVID-19 pandemic preparedness in a small hospital with limited infection prevention staff. ICM for pandemic preparedness was feasible and contributed to an increase in COVID-19 knowledge and comfort.

The disease caused by severe acute respiratory syndrome coronavirus 2 (SARS-CoV-2), coronavirus disease 2019 (COVID-19), has caused a global pandemic straining healthcare systems worldwide.^1,2^ Preparing and responding to any pandemic is challenging. Small and safety-net hospitals may have limited staffing and resources when preparing for or responding to pandemics. Successful strategies in these settings are of practical significance. Small hospitals play a major role in healthcare delivery in the United States: in a recent survey, 70% of hospitals have <200 beds and 10% have <25 beds.^3^ Smaller hospitals also have limited infection preventionist and infectious diseases staff.^4,5^ A survey of the SHEA Research Network showed that smaller hospitals had an average of 1.1 infection preventionists per 100 inpatient census, varying greatly from larger facilities.^5^ These staffing disparities pose a significant challenge for pandemic preparedness. The rapidly evolving needs in a pandemic strain human resources and decrease the availability of healthcare workers (HCWs) and infection preventionists. An inverted classroom methodology (ICM) may help leverage limited human resources during times of significant need, such as pandemic response.

ICM is a blended learning model in which students interact with new material first and subsequently use class time to discuss the new information.^6^ An online self-directed learning phase precedes the instruction phase, where active learning is used to assimilate information and to identify knowledge gaps. ICM has gained traction in medical education, and studies have shown improvements in learning, engagement, and student satisfaction.^6-8^ Faced with COVID-19, our facility used ICM as a major tool for pandemic preparedness to leverage our limited infectious diseases and infection preventionist human resources. We assessed the feasibility of ICM for pandemic preparedness and its impact on knowledge, attitudes, and perceptions of HCWs.

## Methods

We conducted an anonymous cross-sectional survey at a 151-bed urban safety-net community teaching hospital. Our organization has 1,098 employees, of whom 671 work in the hospital building. One infectious diseases physician and one infection preventionist are on staff. Education via ICM started on March 1, 2020. Online videos on COVID-19 topics were created by the infectious diseases physician using Microsoft PowerPoint, and they were hosted on the Vimeo video platform. Privacy settings were set to a private link. Videos could not be found via open search. The link could be shared and viewed multiple times. In-person and virtual town halls occurred after dissemination of the videos. Town halls were led by the infection preventionist and infectious diseases physician and served as the instruction phase, providing opportunities for questions and knowledge application from the videos. Eight town halls occurred each week from March 1 to April 3, 2020. All departments and disciplines were invited. A 30-question survey was conducted to assess HCW attitudes and perceptions about key COVID-19 topics before and after ICM. An e-mail was sent with a link to the online survey on April 6, 2020. The initial e-mail was followed by reminders at 1 and 2 weeks. Responses were submitted anonymously. Respondents were not required to answer every question. Video utilization statistics for the study period were generated. Survey data were collected electronically using Microsoft Forms. N95 respirator use and the COVID-19 census were retrospectively reviewed.

## Results

**Fig. 1.**
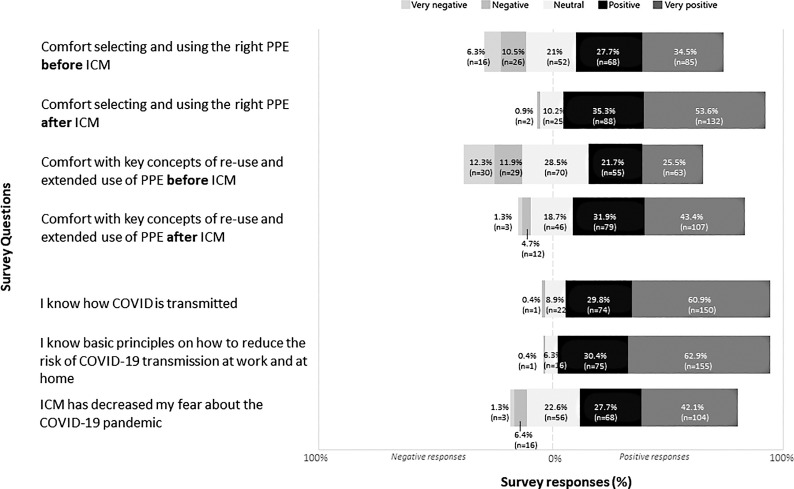
Impact of inverted classroom methodology (ICM) on healthcare worker (HCW) attitudes and perceptions about COVID-19.

In total, 4,312 video plays occurred, of which 2,949 were in a desktop browser, 1,331 were in a mobile phone, and 32 were in a tablet. An average of 67.36 views per day occurred. The most widely seen videos were on personal protective equipment (PPE) use and SARS-CoV2 transmission dynamics (2,784 views), followed by general information about COVID-19 (673 views), COVID-19 triage process (287 views), flattening the curve (236 views), and hospital pandemic plans (202 views). In total, we received 247 responses to the survey, representing multiple hospital disciplines and departments (Table 1). Videos were rated on Likert scales as follows; 78.4% very helpful, 16.7% somewhat helpful, and 4.1% neutral. The impact of ICM on HCW attitudes and perceptions about COVID-19 was mostly rated positive (27.7%–32.5%) or very positive (42.1%–62.9%) on Likert scales (Fig. 1). After ICM, comfort selecting PPE was rated 26.7% more favorably and comfort with extended use and reuse was rated 28% more favorably (Fig. 1). Daily N95 respirator use decreased from a peak daily average of 253 to 93 after education despite an increase in COVID-19 cases from 20% to 55% as a proportion of the daily hospital census.

**Table 1.** Survey Respondents by Department and Discipline

Total Survey Respondents	No. (%) (N=247)
**Distribution by Departments**	
Other nonclinical staff	74 (29.9)
Adult medical, surgical, and critical care units	45 (18.2)
Outpatient care	30 (12.1)
Obstetrics and gynecology units	22 (8.9)
Emergency room	17 (6.8)
Hospital administration	16 (6.4)
Laboratory	10 (4)
Radiology	10 (4)
Pharmacy	9 (3.6)
Pediatrics	8 (3.2)
Sterile processing	3 (1.2)
Operating room	3 (1.2)
**Distribution by Disciplines**	
Other nonclinical professions	76 (30.7)
Nurses	60 (24.2)
Licensed independent practitioners (ie, physicians)	30 (12.1)
Hospital administration	21 (8.5)
Phlebotomy, patient care technician, and laboratory technologist	17 (6.8)
Transportation, telecommunications, or security	14 (5.6)
Radiology technologist	11 (4.4)
Pharmacist or pharmacy technologist	7 (2.8)
Facilities management engineer	7 (2.8)
Environmental services	4 (1.6)

## Discussion

ICM was a feasible and efficient way to deliver educational content for pandemic preparedness at a community hospital with limited infection preventionist and infectious diseases physician staff. The videos were easy to create and convenient to access across different devices at any time. This flexibility was valuable for educators and learners through the challenging time constraints of early pandemic planning. Town halls served as the instruction phase for education and addressed knowledge gaps on topics such as transmission dynamics, guidance for PPE use, reuse, and extended use. These were held in person and via online videoconferencing platforms and captured different disciplines, departments, and shifts.

ICM was well received and a better understanding of transmission dynamics and PPE use was reported in our survey. Understanding selection, use, re-use and extended use concepts may have contributed to a reduction in N95 use despite an increase in COVID-19 cases. A decrease in fear was also reported in our survey. Managing fear and fear contagion is important during pandemics. Factors involved in healthcare worker (HCW) fear during pandemics include fear of becoming infected themselves and fear of infecting family members, friends, and colleagues.^9,10^ Studies during the SARS-CoV-1 outbreak showed that most HCWs felt transmission may not be fully avoidable by complying with or maintaining infection control practices. High stress, heavy workload, and sudden changes in guidelines added to fear, fear contagion, and even post-traumatic stress disorder.^10^ The use of ICM to target high-yield topics on how to stay safe at work and at home may have contributed to this reduction in fear, along with further experience with COVID-19 as the pandemic evolved.

Our approach had its challenges as well. Capturing all staff and shifts for town halls can be difficult. We targeted high-risk departments for in-person sessions, such as emergency rooms, inpatient units, and respiratory technologists. Virtual town halls provided significant flexibility to reach early and late shifts. Our ability to deliver content would be have been much more challenging without ICM and was supplemented further with a train-the-trainer programs.

ICM has been used successfully at various levels of medical and nursing education. This model is now widely used in colleges of medicine nationwide, and ICM is gaining momentum in graduate medical education.^6^ In these settings, ICM was effective in improving knowledge and competence with high student satisfaction.^8^ It has also yielded positive academic outcomes in nursing.^7^ The use of ICM in rapidly changing environments with significant education needs is a natural next step. To our knowledge, this is the first description of its use for pandemic planning and response.

We report our experience implementing ICM at an urban community hospital with limited infection prevention staff. This model was effective, efficient, and well received by staff of various disciplines and departments. Our survey demonstrated a positive impact on knowledge and perceptions on key COVID-19 topics, such as modes of transmission, PPE use, and fear. In a pandemic with rapidly evolving knowledge and education needs, ICM can be an effective education and preparedness tool.
